# Serum Vitamin D, Vitamin D Binding Protein, and Risk of Colorectal Cancer

**DOI:** 10.1371/journal.pone.0102966

**Published:** 2014-07-18

**Authors:** Gabriella M. Anic, Stephanie J. Weinstein, Alison M. Mondul, Satu Männistö, Demetrius Albanes

**Affiliations:** 1 Cancer Prevention Fellowship Program, Division of Cancer Prevention, National Cancer Institute, National Institutes of Health, Bethesda, Maryland, United States of America; 2 Nutritional Epidemiology Branch, Division of Cancer Epidemiology and Genetics, National Cancer Institute, National Institutes of Health, Bethesda, Maryland, United States of America; 3 Department of Chronic Disease Prevention, National Institute for Health and Welfare, Helsinki, Finland; National Health Research Institutes, Taiwan

## Abstract

**Background:**

We previously reported a positive association between serum 25-hydroxyvitamin D (25(OH)D) and colorectal cancer risk. To further elucidate this association, we examined the molar ratio of 25(OH)D to vitamin D binding protein (DBP), the primary 25(OH)D transport protein, and whether DBP modified the association between 25(OH)D and colorectal cancer risk.

**Methods:**

In a nested case-control study within the Alpha-Tocopherol, Beta-Carotene Cancer Prevention Study, controls were 1∶1 matched to 416 colorectal cancer cases based on age and date of blood collection. Logistic regression was used to estimate odds ratios (ORs) and 95% confidence intervals (CI) for quartiles of 25(OH)D, DBP, and the molar ratio of 25(OH)D:DBP, a proxy for free, unbound circulating 25(OH)D.

**Results:**

Comparing highest to lowest quartiles, DBP was not associated with colorectal cancer risk (OR = 0.91; 95% CI: 0.58, 1.42, p for trend  = 0.58); however, a positive risk association was observed for the molar ratio of 25(OH)D:DBP (OR = 1.44; 95% CI: 0.92, 2.26, p for trend  = 0.04). In stratified analyses, the positive association between 25(OH)D and colorectal cancer was stronger among men with DBP levels above the median (OR = 1.89; 95% CI: 1.07, 3.36, p for trend  = 0.01) than below the median (OR = 1.20; 95% CI: 0.68, 2.12, p for trend  = 0.87), although the interaction was not statistically significant (p for interaction  = 0.24).

**Conclusion:**

Circulating DBP may influence the association between 25(OH)D and colorectal cancer in male smokers, with the suggestion of a stronger positive association in men with higher DBP concentrations. This finding should be examined in other populations, especially those that include women and non-smokers.

## Introduction

Vitamin D is thought to protect against carcinogenesis through mechanisms that include promotion of cell differentiation and apoptosis, inhibition of cell proliferation, and modulation of inflammation and immunity [1], [2]. The majority of vitamin D is produced in the skin by exposing 7-dehydrocholesterol to UVB radiation, although it can also be obtained from dietary sources including fatty fish, eggs, fortified dairy and cereal products and supplements. 25-Hydroxyvitamin D (25(OH)D) is the primary form of vitamin D in circulation and is considered the best indicator of an individual's vitamin D status since it is an integrated measure of vitamin D obtained from diet, supplements, and sun exposure [3].

There is some biologic evidence to suggest that vitamin D impacts colon tissue. Both normal and malignant colon epithelium express the 1α-hydroxylase enzyme that converts 25(OH)D to the active hormonal vitamin D metabolite 1,25-dihydroxyvitamin D (1,25(OH)_2_D) [4], [5]. Furthermore, exposing colon cancer cells to 1,25(OH)_2_D promotes cellular differentiation [6] and a higher serum concentration of 25(OH)D is linked to lower rates of colorectal epithelial cell proliferation [7]. Meta-analyses that include up to 10 nested-case-control studies found a modest yet significant inverse association between pre-diagnostic blood concentrations of 25(OH)D and colorectal cancer risk [8]–[11]. Not all studies have supported this inverse association, however. For example, we previously reported a positive association between serum 25(OH)D and risk for colon and rectal cancers in the Alpha-Tocopherol, Beta-Carotene Prevention (ATBC) Study [12], and two randomized clinical trials showed no effect of vitamin D supplementation on colorectal cancer incidence [13], [14]. In addition, a study that examined genetic predictors of circulating 25(OH)D concentrations did not find an association between these genetic variants and colorectal cancer [15].

Vitamin D binding protein (DBP) is the primary transport protein of 25(OH)D in circulation [16], with approximately 88% of 25(OH)D being bound to DBP and most of the rest bound to albumin, leaving less than 1% of 25(OH)D in an unbound, “free” state [16]. In addition to being a carrier for vitamin D metabolites, DBP also has anti-inflammatory and immunoregulatory functions [17]–[20], and may play a role in several chronic disease outcomes [21]. In the ATBC Study, serum DBP has been shown to modify 25(OH)D risk associations with pancreatic [22], prostate [23], and bladder cancers [24], and was inversely related to pancreatic [22] and renal cell carcinoma [25], supporting the idea that DBP quantification could provide a more complete understanding of the association between 25(OH)D and cancer risk.

We conducted a nested case-control study within the ATBC Study to investigate whether circulating DBP or the 25(OD)D:DBP molar ratio were associated with colorectal cancer risk, and whether DBP modified the positive association with serum 25(OH)D previously observed in this population [12].

## Materials and Methods

### Study population

The ATBC Study, which has been described previously [26], was a randomized, double-blind, placebo controlled, primary chemoprevention trial, that involved daily supplementation with α-tocopherol (50 mg/day), β-carotene (20 mg/day), both supplements, or placebo (ClinicalTrials.gov Identifier: NCT00342992). A total of 29,133 men who were residents of southwestern Finland, aged 50–69 years, and who smoked ≥5 cigarettes per day were enrolled into the trial between 1985 and 1988. Study supplementation continued for 5–8 years (median, 6.1 years) until death or trial closure on April 30, 1993. The study was approved by the institutional review boards of the National Cancer Institute and the National Public Health Institute of Finland. Written informed consent was obtained from each participant prior to randomization.

### Case identification and control selection

This study was based on a prior nested case-control analysis of serum 25(OH)D that included 428 colorectal cases and 428 controls matched to cases on age (±1 year) and date of serum collection (±30 days), and who were alive and cancer free at the time the case was diagnosed [12]. Of these, 416 cases and their matched controls had serum available for the DBP assay. All cases were identified by the Finnish Cancer Registry, which provides nearly 100% incident case ascertainment for this cohort [27]. Cases in the current analysis included 231 cancers of the colon and 185 cancers of the rectum, defined by the International Classification of Diseases 9 as codes 153 and 154, respectively, and diagnosed through April 20, 2005.

### Data collection

At baseline, participants completed a questionnaire to obtain information about general risk factors, smoking, medical history, family history of cancer, and vitamin supplement use. A validated food frequency questionnaire was also administered to measure usual consumption, portion size, and frequency of 276 food items and mixed dishes during the previous 12 months [28]. A National Food Composition Database from the National Public Health Institute of Finland was used to estimate nutrient intake [29].

### Laboratory measures

Fasting serum samples were collected at the pre-randomization baseline visit and stored at −70°C. Concentrations of 25(OH)D were previously measured in matched case-control sets [12] by Heartland Assays, Inc. (Ames, IA, USA), using the DiaSorin Liaison 25(OH)D TOTAL assay [30], [31]. Each batch included four or six quality control samples that came from an ATBC serum pool or standard reference materials provided by the National Institute of Standards and Technology (NIST) [32]. Nested components-of-variance analysis [33] was used to calculate the interbatch and intrabatch coefficients of variation that were 7.1% and 10.1%, respectively, for the NIST and ATBC serum pool combined.

Vitamin D binding protein was measured using the Quantikine Human Vitamin D Binding Protein Immunoassay kit (Catalog number DVDVP0, R&D Systems, Inc., Minneapolis, MN, USA) at the Clinical Support Laboratory, SAIC-Frederick, Inc., Frederick National Laboratory for Cancer Research (Frederick, MD, USA). Blinded quality control samples were included in each batch and comprised approximately 10% of the total samples. Interbatch and intrabatch coefficients of variation were 10.8% and 15.2%, respectively.

### Statistical analysis

Medians (for continuous data) and proportions (for categorical data) of baseline characteristics were calculated among the controls by quartile of DBP. Kruskal-Wallis and chi-square tests were used to assess the statistical significance of differences across DBP quartiles. Conditional logistic regression was conducted to estimate odds ratios (ORs) and corresponding 95% confidence intervals (CI) for the risk of colorectal cancer by quartile of 25(OH)D, DBP, and the molar ratio of 25(OH)D:DBP, an estimation of free circulating 25(OH)D [34], [35]. Since 25(OH)D levels fluctuate throughout the year, season-specific quartiles of 25(OH)D were calculated based on the distribution of 25(OH)D in controls, split by season of blood draw during darker months (November-April) or sunnier months (May-October) and then merged into one variable. Quartiles of DBP and the molar ratio of 25(OH)D:DBP were determined based on the distribution of each variable in the controls. To test for linear trend, a term with the median values of the main effect was entered into the model as a continuous ordinal variable. Models were conditioned on the matching factors of age and date of blood collection.

The variables presented in Table 1 were assessed as potential confounders by entering each into an age-adjusted model to determine if the effect estimate for DBP changed more than 10%. None of the factors significantly altered the effect estimate at that level; therefore, the final multivariable model adjusted for factors included in our earlier analysis of 25(OH)D and colorectal cancer [12] (i.e., years of smoking, and serum α-tocopherol, β-carotene, and retinol) and known colorectal risk factors (i.e., BMI, height, and physical activity). We also present results for the multivariable model of 25(OH)D adjusted for DBP, and vice versa.

**Table 1:** Select baseline characteristics of controls by quartile of serum vitamin D binding protein, ATBC Study, 1985-2004.

	Quartile of serum vitamin D binding protein (nmol/L)				
Baseline characteristic^1^	Q1≤4369	Q2 4370–<5579	Q3 5579–<6993	Q4 ≥6993	p-value
N	104	104	104	104	
Age, y	58	57	57	58	0.84
Height, cm	175	174	173	174	0.23
Weight, kg	76.8	79.3	76.2	76.4	0.46
Body mass index, (kg/m^2^)	25.4	26.3	25.3	25.9	0.22
Education, % >elementary	21.2	26.9	22.1	19.2	0.59
Cigarettes/day	20.0	19.0	20.0	20.0	0.53
Total years smoked	36.5	36.0	36.0	38.0	0.92
Physical activity (% that reported light or moderate work activity and at least moderate leisure activity)	23.1	24.0	19.2	23.1	0.85
Family history of colorectal cancer, % yes	3.9	1.9	2.9	2.9	0.88
Energy intake, kcal/day^2^	2,704	2,556	2,617	2,578	0.28
Vitamin D supplement use, % yes^2^	13.5	7.7	5.8	3.9	0.06
Dietary vitamin D intake, µg/day^2^	5.0	4.5	4.5	4.7	0.42
Supplemental vitamin D intake, µg/day^2^	8.6	5.2	3.0	1.5	0.02
Calcium supplement use, % yes^2^	14.4	9.6	8.7	8.7	0.46
Total calcium intake (diet and supplements), mg/day^2^	1,342	1,353	1,377	1,338	0.98
Total fat intake, g/day^2^	124	115	116	116	0.2.5
Ethanol intake, g/day^2^	12.5	10.7	8.4	11.4	0.52
Season at blood draw, % May-October	43.3	35.6	32.7	31.7	0.29
Serum biomarkers					
DBP, nmol/L	3,555	5,004	6,188	8,120	<0.0001
25(OH)D, nmol/L	31.1	31.7	28.6	31.9	0.63
25(OH)D:DBP molar ratio^3^ (x 10^3^)	9.0	6.6	5.1	4.0	<0.0001
α-Tocopherol, mg/L	11.6	11.7	11.3	11.3	0.91
β-Carotene, µg/L	197	204	181	168	0.28
Total cholesterol, mmol/L	6.1	6.3	6.4	6.3	0.18
High-density lipoprotein cholesterol, mmol/L	1.2	1.1	1.1	1.2	0.23
Retinol, µg/L	557	604	583	573	0.30

1 All values are medians or proportions.

2 Dietary and supplement intake data were available for 95% of subjects.

3 A proxy for free circulating 25(OH)D.

To evaluate effect modification, models for 25(OH)D and the molar ratio of 25(OH)D:DBP were stratified by the median DBP value and models for DBP were stratified by the median value of season-specific 25(OH)D. We also evaluated DBP stratified by α-tocopherol and β-carotene trial supplementation status, follow-up time (≤10 and >10 years), and the median split value of age, BMI, and dietary calcium intake. Unconditional logistic regression, adjusted for the matching factors of age and date of blood collection, was used for stratified models to maintain subjects that were not in the same stratum as their matched case or control. The results did not appreciably change when unconditional models were used instead of conditional logistic regression, therefore, this method should not bias the findings. To test for effect modification, a cross-product term of the main effect (25(OH)D, 25(OH)D:DBP molar ratio, and DBP) in quartiles and the effect modifier split at the median or as a two-level categorical variable was included in a model that adjusted for the matching factors of age at randomization and time of blood draw.

Statistical analyses were done using SAS 9.2 (SAS Institute, Cary, NC). All p-values were two-sided.

## Results

Baseline characteristics of the controls across quartiles of DBP are presented in Table 1. Age, BMI, education, cigarette use, physical activity, calcium and fat intake, and baseline serum concentrations of α-tocopherol, β-carotene, cholesterol, and retinol were similar across the quartiles. Men with higher serum DBP concentrations had a significantly lower molar ratio of 25(OH)D:DBP (p = <0.0001) and lower vitamin D intake from supplements (p = 0.02).

Serum DBP was inversely correlated with the molar ratio of 25(OH)D:DBP (Spearman correlation coefficient (*r*)  = −0.44, p = <0.0001), but not strongly correlated with serum 25(OH)D (*r* = 0.08, p = 0.02). No correlations were observed between serum DBP and cigarettes smoked per day (*r* = 0.05, p = 0.14), baseline serum α-tocopherol (*r* = 0.006, p = 0.85) and β-carotene (*r* = −0.05, p = 0.12), age at randomization (*r* = 0.02, p = 0.62), BMI (*r* = 0.05, p = 0.18), or daily intake of vitamin D (diet and supplements) (*r* = −0.04, p = 0.26), calcium (*r* = −0.02, p = 0.57), fat (*r* = −0.02, p = 0.57), alcohol (*r* = 0.05, p = 0.20), or energy (*r* = −0.01, p = 0.79). Weak correlations were observed between DBP concentration and serum retinol (*r* = 0.13, p = 0.0002) and total cholesterol (*r* = 0.07, p = 0.04).

Similar to our previous findings [12], higher serum 25(OH)D was associated with an increased risk of colorectal cancer (OR  = 1.53; 95% CI: 1.01, 2.32 for highest versus lowest quartile, p for trend  = 0.04) and further adjustment for DBP did not alter the positive association between 25(OH)D and colorectal cancer (Table 2). Circulating DBP was not associated with risk of colorectal cancer (OR  = 0.91; 95% CI: 0.58, 1.42, p for trend  = 0.58), and further adjustment for 25(OH)D did not alter this association. Null associations with DBP were also observed for proximal and distal colon cancers and when colon and rectal cancers were analyzed separately (data not shown). A positive association was observed between the molar ratio of 25(OH)D:DBP, a proxy for free circulating 25(OH)D, and colorectal cancer risk (OR  = 1.44; 95% CI: 0.92, 2.26 for highest versus lowest quartile, p for trend  = 0.04), although only the OR for the third quartile was statistically significant. Non-significant positive associations with the molar ratio of 25(OH)D:DBP were also observed in sub-analyses of proximal and distal colon cancer and colon and rectal cancers (data not shown).

**Table 2:** Association between serum 25(OH)D, DBP, and the 25(OH)D:DBP molar ratio, and the risk of colorectal cancer, ATBC Study.

	Quartile 1	Quartile 2	Quartile 3	Quartile 4	
	OR (95% CI)	OR (95% CI)	OR (95% CI)	OR (95% CI)	p-trend
**25(OH)D**					
Range (nmol/L)^1^					
No. Cases/no. controls	93/106	91/104	111/103	121/103	
Model 1^b^	Reference	1.02 (0.69, 1.50)	1.22 (0.83, 1.80)	1.35 (0.91, 2.01)	0.11
Model 2^c^	Reference	1.06 (0.71, 1.59)	1.29 (0.86, 1.92)	1.53 (1.01, 2.32)	0.04
Model 3^d^	Reference	1.05 (0.70, 1.58)	1.28 (0.85, 1.92)	1.56 (1.02, 2.36)	0.03
**DBP**					
Range (nmol/L)	≤4369	4370–<5579	5579–<6993	≥6993	
No. Cases/no. controls	100/104	116/104	104/104	96/104	
Model 1^2^	Reference	1.14 (0.78, 1.66)	1.02 (0.68, 1.55)	0.94 (0.61, 1.45)	0.65
Model 2^3^	Reference	1.09 (0.74, 1.60)	1.01 (0.66, 1.56)	0.91 (0.58, 1.42)	0.58
Model 3^4^	Reference	1.06 (0.71, 1.56)	0.95 (0.62, 1.47)	0.85 (0.54, 1.33)	0.40
**25(OH)D:DBP molar ratio (x10** 3 **)**					
Range	≤3.5	3.6–<5.3	5.3–<9.1	≥9.1	
No. Cases/no. controls	88/104	75/104	148/104	105/104	
Model 1^2^	Reference	0.85 (0.56, 1.29)	1.83 (1.22, 2.73)	1.29 (0.84, 1.99)	0.09
Model 2^3^	Reference	0.86 (0.57, 1.32)	1.96 (1.29, 2.97)	1.44 (0.92, 2.26)	0.04

Conditional logistic regression was used for all models. OR  =  odds ratio; CI  =  confidence interval.

1 Cut-points for season specific quartiles (nmol/L): winter  = Q1: ≤18.3, Q2: >18.3–≤26.9, Q3: >26.9–≤42.0, Q4: >42.0; summer  = Q1: ≤27.0, Q2: >27.0 and ≤38.7, Q3: >38.7–≤53.4, Q4: >53.4.

2 Conditioned on the matching factors age at randomization and date of blood collection.

3 Conditioned on the matching factors age at randomization and date of blood collection and adjusted for years of smoking, serum α-tocopherol, serum β-carotene, serum retinol, BMI, height, and physical activity.

4 Adjusted for factors in model 2 with additional adjustment for quartiles of 25(OH)D or DBP.

In stratified analyses (Table 3), the positive association between 25(OH)D and colorectal cancer was evident primarily among men with higher circulating DBP (OR  = 1.89; 95% CI: 1.07, 3.36 for highest versus lowest quartile, p for trend  = 0.01) than men with lower DBP levels (OR  = 1.20; 95% CI: 0.68, 2.12, p for trend  = 0.87), although the interaction was not statistically significant (p for interaction  = 0.24). 25(OH)D concentration did not significantly modify the association between circulating DBP and colorectal cancer risk (p for interaction  = 0.17), although there was suggestion of an inverse association with DBP in men with low 25(OH)D levels (OR  = 0.70; 95% CI: 0.39, 1.26 for highest versus lowest quartile, p for trend  = 0.19) and a statistically non-significant positive association with DBP in men with higher levels of 25(OH)D (OR  = 1.24; 95% CI: 0.70, 2.18 for highest versus lowest quartile, p for trend  = 0.63). Similar to the finding for 25(OH)D, a positive association between the molar ratio of 25(OH)D:DBP and colorectal cancer risk was observed for men with circulating DBP concentrations above the median (OR  = 1.55; 95% CI: 0.76, 3.13 for highest versus lowest quartile, p for trend  = 0.01), while the ratio was not related to risk in men with lower DBP levels (OR  = 0.96; 95% CI: 0.50, 1.82 for highest versus lowest quartile, p for trend  = 0.89); the test for interaction was marginally not statistically significant (p for interaction  = 0.07).

**Table 3:** Odds ratios and 95% confidence intervals for the association between serum 25(OH)D, DBP, and the 25(OH)D:DBP molar ratio and colorectal cancer risk in stratified models, ATBC Study.

	Quartile 1	Quartile 2	Quartile 3	Quartile 4		
	OR (95% CI)	OR (95% CI)	OR (95% CI)	OR (95% CI)	p-trend	p-interaction
**25(OH)D**						
Range (nmol/L)^1^						
DBP <median						
No. Cases/no. controls	54/57	51/49	60/53	51/49		
Model 1^2^	Reference	1.10 (0.64, 1.89)	1.20 (0.71, 2.04)	1.12 (0.65, 1.94)	0.98	0.24
Model 2^3^	Reference	1.10 (0.63, 1.91)	1.26 (0.73, 2.15)	1.20 (0.68, 2.12)	0.87	
Model 3^4^	Reference	1.07 (0.61, 1.87)	1.24 (0.72, 2.13)	1.17 (0.66, 2.08)	0.91	
DBP ≥median						
No. Cases/no. controls	39/49	40/55	51/50	70/54		
Model 1^2^	Reference	0.92 (0.51, 1.64)	1.28 (0.72, 2.28)	1.63 (0.94, 2.83)	0.02	
Model 2^3^	Reference	1.05 (0.57, 1.92)	1.39 (0.77, 2.51)	1.89 (1.07, 3.36)	0.01	
Model 3^4^	Reference	1.05 (0.57, 1.93)	1.39 (0.77, 2.51)	1.91 (1.07, 3.40)	0.01	
**DBP**						
Range (nmol/L)	≤4369	4370–<5579	5579–<6993	≥6993		
25(OH)D <median						
No. Cases/no. controls	56/52	49/54	41/55	38/49		
Model 1^2^	Reference	0.84 (0.49, 1.45)	0.70 (0.40, 1.22)	0.71 (0.40, 1.27)	0.20	0.17
Model 2^3^	Reference	0.80 (0.46, 1.40)	0.67 (0.38, 1.18)	0.70 (0.39, 1.26)	0.19	
Model 3^4^	Reference	0.80 (0.45, 1.39)	0.66 (0.37, 1.17)	0.69 (0.38, 1.25)	0.19	
25(OH)D ≥median						
No. Cases/no. controls	44/52	67/50	63/49	58/55		
Model 1^2^	Reference	1.59 (0.92, 2.73)	1.53 (0.88, 2.66)	1.26 (0.73, 2.18)	0.58	
Model 2^3^	Reference	1.54 (0.88, 2.69)	1.48 (0.84, 2.60)	1.24 (0.70, 2.18)	0.63	
Model 3^4^	Reference	1.53 (0.87, 2.68)	1.46 (0.83, 2.57)	1.22 (0.69, 2.16)	0.68	
**25(OH)D:DBP molar ratio (x10** 3 **)**						
Range	≤3.5	3.6–<5.3	5.3–<9.1	≥9.1		
DBP <median						
No. Cases/no. controls	29/26	36/43	69/57	82/82		
Model 1^2^	Reference	0.75 (0.38, 1.49)	1.10 (0.58, 2.08)	0.92 (0.49, 1.72)	0.94	0.07
Model 2^3^	Reference	0.78 (0.38, 1.60)	1.17 (0.61, 2.25)	0.96 (0.50, 1.82)	0.89	
DBP ≥median						
No. Cases/no. controls	59/78	39/61	79/47	23/22		
Model 1^2^	Reference	0.85 (0.50, 1.43)	2.24 (1.36, 3.69)	1.40 (0.71, 2.77)	0.02	
Model 2^3^	Reference	0.86 (0.50, 1.48)	2.50 (1.49, 4.19)	1.55 (0.76, 3.13)	0.01	

Unconditional logistic regression was used for all models. OR  =  odds ratio; CI  =  confidence interval.

1 Cut-points for season specific quartiles (nmol/L): winter  = Q1: ≤18.3, Q2: >18.3–≤26.9, Q3: >26.9–≤42.0, Q4: >42.0;

Summer  = Q1: ≤27.0, Q2: >27.0 and ≤38.7, Q3: >38.7–≤53.4, Q4: >53.4.

2 Model adjusted for matching factors of age at randomization and date of blood collection.

3 Model adjusted for age at randomization, date of blood collection, years of smoking, serum α-tocopherol, serum β-carotene, serum retinol, BMI, height, and physical activity.

4 Adjusted for same factors in model 2, with additional adjustment for quartiles of 25(OH)D or DBP.

Other factors, including age, BMI, α-tocopherol and β-carotene trial supplementation, calcium intake, and follow-up time did not significantly modify the association between serum DBP and colorectal cancer risk (data not shown). In models stratified by follow-up time, the positive association between the 25(OH)D:DBP molar ratio and risk of colorectal cancer was somewhat attenuated for cases diagnosed more than 10 years after baseline blood collection (OR  = 1.11; 95% CI: 0.67, 1.83) as compared with cases diagnosed earlier (OR  = 1.66; 95% CI: 0.79, 3.50) (p for interaction  = 0.06).

## Discussion

Overall, we did not observe an association between circulating DBP and risk of colorectal cancer, and as we previously reported [12], serum 25(OH)D was positively associated with colorectal cancer. The positive association with 25(OH)D appeared stronger among men with a higher serum concentration of DBP, although the interaction was not formally statistically significant. Also, men with a higher 25(OH)D:DBP ratio, a proxy for free circulating 25(OH)D [34], [35], appeared to be at an increased risk of colorectal cancer, with stronger risk estimates for men with a serum DBP concentration above the median.

Our finding that serum 25(OH)D is positively associated with colorectal cancer risk contrasts with data from meta-analyses supporting an inverse association between serum 25(OH)D status and colorectal cancer [8]–[11]. Although as yet unexplained, the positive association that we observe for higher serum 25(OH)D in the ATBC Study is potentially due to the fact that all men in ATBC were smokers, and smoking may alter the physiological response to 25(OH)D status: experimental evidence shows that smoking inactivates the physiologically active metabolite 1,25(OH)_2_D by increasing expression of the *CYP24A1* gene which works to catabolize 1,25(OH)_2_D [36]. It is not known whether DBP-bound or free circulating vitamin D is more relevant to colorectal cancer risk. According to the ‘free hormone hypothesis’, only unbound circulating hormones are bioavailable and physiologically active [37]. Our finding of similar positive associations for total serum 25(OH)D and the molar ratio of 25(OH)D:DBP, and weaker associations between total 25(OH)D and risk in the presence of lower DBP concentration, which may be indicative of lower free circulating vitamin D levels, suggest that free circulating vitamin D is not more strongly associated with colorectal cancer as postulated by the free hormone hypothesis, and leaves open other biological mechanisms of action.

DBP has several biological functions in addition to its role as a carrier for circulating vitamin D metabolites. It serves as an actin scavenger, binding to globular actin released from tissue injury or death, thereby preventing filamentous actin in circulation and vascular occlusion [16], [18]. DBP is also involved in immune response when deglycosylating enzymes convert DBP to a macrophage-activating factor that has anti-angiogenic and anti-tumorigenic properties [19], [38], and that plays a role in promoting neutrophil chemotaxis during inflammation [17], [18]. In the current study, we did not observe a main effect association with circulating DBP concentrations, suggesting that these other functions of DBP may not influence colorectal carcinogenesis.

Cellular uptake of DBP bound 25(OH)D occurs when the cell surface receptors megalin, cubilin and Dab2 bind to and endocytose the DBP-25(OH)D complex [39]. Colon epithelial cells express both megalin and Dab2 [40], facilitating entry of DBP-bound 25(OH)D into colon tissue. Although speculative, the stronger positive risk association that we observed with 25(OH)D and the 25(OH)D:DBP ratio in men with higher DBP concentration may be due to increased uptake of DBP bound 25(OH)D into colon tissue in the presence of high DBP concentration. Megalin is a multi-ligand plasma membrane receptor that supports endocytosis of several protein-bound lipid soluble molecules in addition to the DBP-25(OH)D complex [41], all of which compete for cellular uptake by available megalin receptors. For example, testosterone bound to sex hormone-binding globulin (SHBG), another megalin ligand, is associated with reduced risk of colorectal cancer in men [42]. It is possible that in the presence of higher DBP concentration, less SHBG-bound testosterone is taken up by colon cells, thereby reducing its protective effects in colorectal carcinogenesis. Higher DBP concentration may also upregulate megalin mediated endocytosis of other substances that could directly influence colon cancer risk; e.g., smoking-related carcinogens such as the nitrosamine NNK that have been shown to stimulate colon cancer cell growth and metastasis [43], [44].

We are not aware of other studies that have examined the association between DBP and colorectal cancer risk. DBP was not associated with prostate cancer in two nested case-control studies that measured pre-diagnostic DBP [45] and the 25(OH)D:DBP molar ratio (reported as “free 25-D” in the paper) [46]. In the ATBC Study, DBP concentrations were not associated with bladder [24] or prostate [23] cancer, however, an inverse association was observed between DBP levels and risk of pancreatic [22] and renal cell carcinoma [25]. Also in ATBC, DBP concentrations modified the association between serum 25(OH)D and risk of prostate [23], pancreatic [22], and bladder [24] cancers, with higher 25(OH)D being associated with an increased risk of prostate cancer among men with higher DBP concentration and an increased risk of pancreatic cancer in men with lower DBP concentration. For bladder, the inverse association with 25(OH)D was only observed in men with lower concentrations of DBP. It is plausible that the divergent interactions with DBP reflect underlying site-specific biologic mechanisms related to vitamin D status, transport, or cellular uptake that will require replication and further mechanistic elucidation.

Strengths of this study include the measurement of DBP and 25(OH)D in pre-diagnostic fasting serum, a large sample of incident colorectal cancer cases, detailed assessment of potential confounding factors, and up to 20 years of follow-up. The study is limited by only including male smokers, which may limit the generalizability of the findings to women and non-smokers. Smoking intensity and duration did not likely confound the results, however, given that DBP was not correlated with these variables, and including them in the multivariable models did not alter the associations. Other limitations include limited statistical power to conduct stratified analyses and the possibility that significant associations were the result of chance due to multiple testing. Another potential limitation is that measuring DBP and 25(OH)D from a single blood sample collected at baseline may not be representative of typical concentrations over time, although circulating DBP appears stable over periods of 1–3 years [47] and has negligible seasonal variability [34]. Although serum 25(OH)D fluctuates by season, there is evidence that 25(OH)D levels remain fairly stable over time [47]–[49]. We cannot rule out, however, that the attenuated positive association between the 25(OH)D:DBP molar ratio and colorectal cancer risk among cases diagnosed more than 10 years after the baseline blood collection was due to individual changes in 25(OH)D concentrations over time.

In summary, although overall DBP concentrations were not associated with risk of colorectal cancer, the positive association between 25(OH)D and the 25(OH)D:DBP molar ratio and colorectal cancer risk appeared somewhat stronger in men with higher DBP concentration. Our findings suggest that DBP, the primary transport carrier protein of vitamin D, may play a role in the association between serum concentration of 25(OH)D and colorectal cancer risk and warrants further examination in populations that include women and non-smokers.
